# Media and suicidal behaviour

**DOI:** 10.1192/j.eurpsy.2021.163

**Published:** 2021-08-13

**Authors:** U. Hegerl

**Affiliations:** Department Of Psychiatry, Psychosomatics, And Psychotherapy, Goethe University Frankfurt, Frankfurt a.M., Germany

## Abstract

Media coverage of suicidal behaviour can induce copycat suicides. This has been clearly confirmed by analysis of suicides following the huge media coverage of the railway suicide of the German national goal keeper in 2009. A socalled ‘Werther effect’ was not only visible in Germany, but also in neighbouring countries (1). Even more disturbing is the fact that these effects were not only short-lived, but a higher number of railway suicides was observed compared to baseline over a two year period (2). Increased cognitive availability of railway suicides might explain this finding. It adds to the important discussion concerning the risks and benefits of public antisuicidal campaigns. Destigmatisaton and normalisation of suicidal behaviour will on the one hand, facilitate helpseeking behaviour of people at risk, but on the other hand, will lower the threshold for committing suicide. Even when the wording within a antisuicide campaign is in line with recomendations of media guidelines, secondary reporting e.g. within social media will not be controllable. Social media are likely to contribute to the spreading and the choice of more lethal suicide methods, as has been shown for carbon monoxide poisoning and poisoning by other gasses (3). An increase of knowledge about and access to more lethal poisoning methods will have a major impact on suicide rates. 1) KOBURGER et al (2015), J Affect Disord 185:38-46 2) HEGERL et al (2013), J Affect Disord. 146: 39-44. 3) PAUL et al (2017), PLoS One 12: e0190136.

**Disclosure:**

No significant relationships.

